# Evaluation of the effects of irrigation and fertilization on tomato fruit yield and quality: a principal component analysis

**DOI:** 10.1038/s41598-017-00373-8

**Published:** 2017-03-23

**Authors:** Xiukang Wang, Yingying Xing

**Affiliations:** 1 College of Life Science, Yan’an University, Yan’an, Shaanxi 716000 China; 2 Institute of Soil and Water Conservation, Chinese Academy of Sciences and Ministry of Water Resources, Yangling, Shaanxi 712100 China

## Abstract

Irrigation and fertilization are key practices for improving the fruit quality and yield of vegetables grown in greenhouses. We carried out an experiment in a solar greenhouse spanning three consecutive growing seasons to evaluate the effects of irrigation and fertilization on the fruit yield and quality, water use efficiency (WUE) and fertilizer partial factor productivity (PFP) of tomatoes. Interactions between irrigation and fertilization treatments and individual factors of irrigation and fertilization significantly (*p* < 0.01) affected fruit yield, WUE and PFP. WUE and fruit yield and quality were more sensitive to changes in irrigation than to changes in fertilizer, but PFP showed the opposite trend. Interestingly, the treatment with moderate irrigation (W2: 75% *ET*_*0*_) and high fertilizer level (F1: 240N−120P_2_O_5_−150K_2_O kg ha^−1^) was twice ranked first after a combinational evaluation. In conclusion, the proper application of drip fertigation (W2F1) may be a good compromise for solar greenhouse-grown tomatoes with regard to fruit yield and quality, WUE, and PFP. The present study sheds light on the contributions of these practices, clarifies their impacts, and provides a basis for evaluating and selecting better management practices for growing greenhouse vegetables.

## Introduction

The management of water and nutrients applied in fertilizers are the two major factors affecting crop growth and productivity^1, 2^. Indeed, crop yield and quality are very sensitive to appropriate water and nutrient contents in the root zone of plants, which can improve the absorbing area and capacity of roots^3^. Sustainable water and fertilizer used in agriculture has become a priority, along with the adoption of field management strategies that maintain satisfactory yields, thus improving both fertilizer and water use efficiency (WUE). In recent years, tomatoes have rapidly become one of the most popular items of produce in the world, and tomatoes are marketed to consumers as a healthy food that can help reduce the risk of contracting certain human diseases and developing many forms of cancer. Tomatoes are one of the most important annual crops in solar greenhouse production systems and also have a high water demand, requiring irrigation throughout the growing seasons^4^. However, in areas of water scarcity, such as northwest China, maximizing water conservation may be more profitable than maximizing crop yield. The WUE in this area is relatively low. Consequently, appropriate irrigation technologies must be selected to maximize the WUE and profits^5^. The process of crop water use has two main components: evaporative losses from the soil and the crop, usually called evapotranspiration, and all the losses resulting from the distribution of the water to the land^6^. In this regard, drip irrigation has contributed greatly to improving WUE because of its ability to supply frequent and small amounts of water that are applied directly to the plant’s root zone, which significantly reduces evapotranspiration and potentially increases production^7, 8^. Among the water management practices for increasing WUE, drip irrigation systems have been widely used in recent years.

Crop productivity is dependent on soil nutrient contents, which usually limit plant yields in agricultural systems^9^. However, the relatively low price of chemical fertilizers and the current prevailing attitude toward increasing fertilizer application to increase crop yield may lead to excessive fertilization. To increase farmers’ incomes, the most effective way to save water and reduce fertilizer input is to improve the WUE and fertilizer partial factor productivity (PFP). PFP (the ratio of crop yield/amount of nutrient applied) is a more appropriate index than other commonly used methods for comparing nutrient use efficiency^10–13^. Specifically, excessive fertilizer input is a common phenomenon and has become a serious threat to the sustainable development of solar greenhouse vegetable production in China, and PFP is an indicator of the degree to which different methods of fertilizer input are effective in decreasing the pollution of groundwater with fertilizers^11^. Water is an important factor in the fate and transport of soil nutrients and in their absorption and utilization by crops, and appropriate water and fertilizer application are simultaneously considered in irrigation and fertilization strategies using drip irrigation. Several studies have investigated the effects of the interactions between irrigation and fertilization on crop production, WUE, and fertilizer leaching^10–13^. At present, the most common view is that the combined effects of well-managed water and fertilizer application can sustain crop productivity. Yields are increased by better matching the temporal and spatial distribution of the water and nutrient supplies during the periods of plant growth with the greatest demand^14, 15^. Another issue to be addressed in ‘precision agriculture’ is regarding the levels of water and nutrients that need to be applied. It is also necessary to determine what strategies best synchronize the management of irrigation and fertilization to improve crop yield and quality, WUE, and fertilizer use efficiency.

Technologies such as drip irrigation can maintain or increase yields while improving WUE and fertilizer use efficiency and decreasing losses that lead to environmental pollution^16^. Ozbahce *et al*.^17^ reported that the optimum fertilization rate for maximum crop yield was the same as that for irrigation treatments. Therefore, appropriate irrigation and fertilization methods are of the greatest importance in increasing the efficiency of water and fertilizer use and reducing the risk of environmental pollution^18, 19^. Several studies have been conducted on how to use irrigation in combination with fertilization as efficiently as possible to maximize profits and reduce groundwater pollution^5, 20^. The effects of water and fertilizer application on crop yield and related parameters such as nitrogen use efficiency, WUE and fruit quality have been reported^21–25^. Little information is available on the best integrated management practices relating to the precise irrigation and fertilization rates needed to achieve a high yield of tomatoes, a high WUE and better fruit quality, as well as lower levels of water and fertilizer input.

It is unclear how much water and fertilizer must be applied to a sustain tomato productivity and fruit quality while effectively managing water and fertilizer output and which statistical and analytical methods should be used to assess the results. Consequently, a more accurate method is needed to estimate the degree of influence of management practices on the effects of irrigation and fertilization on tomato quality and yield, WUE, and efficient fertilizer use. The aims of this study were (i) to analyse the effect of the amount of irrigation in combination with the fertilizer supply on tomato quality and yield, WUE and PFP and (ii) to evaluate and select better management practices for growing tomatoes in solar greenhouses using a principal component analysis (PCA).

## Results

### Effects of irrigation and fertilization on the yield, WUE and PFP at different water and fertilizer input levels

**Figure 1:**
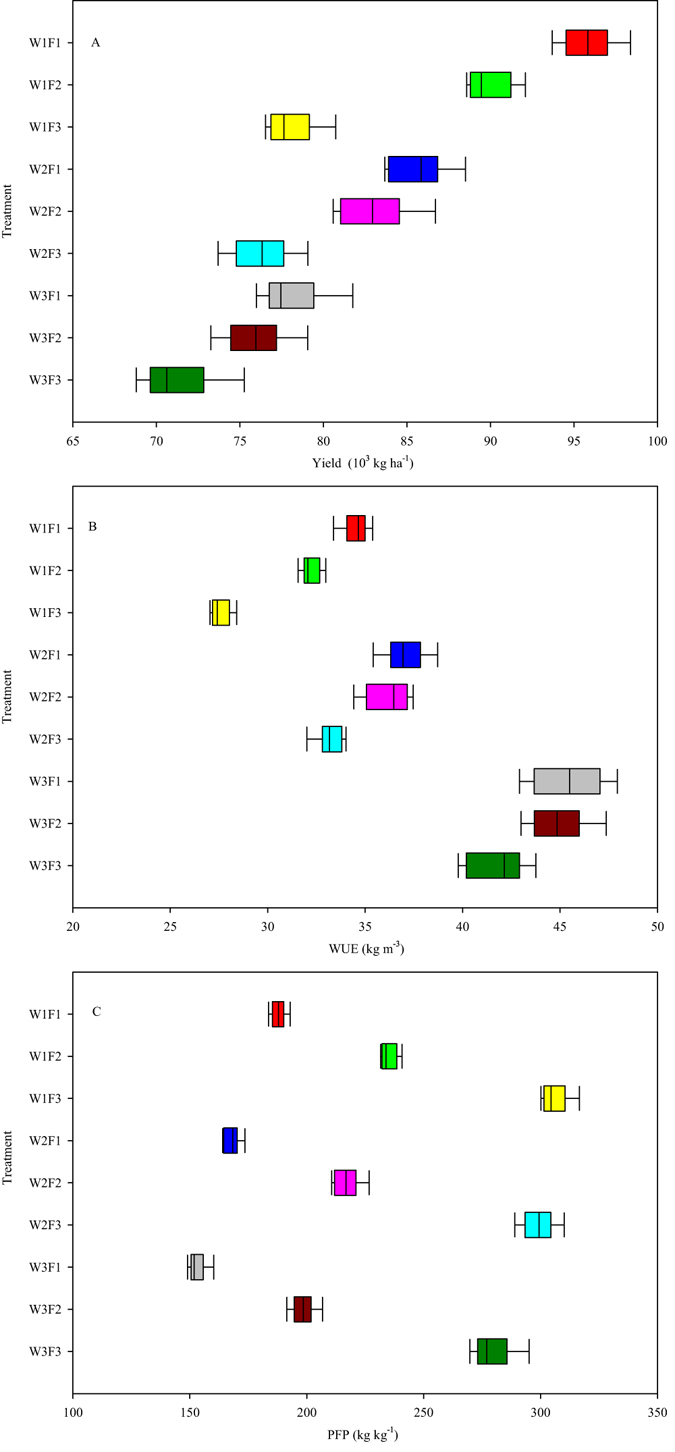
Effects of irrigation and fertilization treatments on fruit yield (**A**), water use efficiency (WUE) (**B**) and partial factor productivity (PFP) (**C**) in three consecutive growing seasons in 2012−2013. The treatments are noted as W1, 100% *ET*_*0*_; W2, 75% *ET*_*0*_; W3, 50% *ET*_*0*_; F1, N240−P_2_O_5_120−K_2_O150 kg ha^−1^; F2, N180−P_2_O_5_90−K_2_O112.5 kg ha^−1^; and F3, N120−P_2_O_5_60−K_2_O75 kg ha^−1^. Boxes show the 25th and 75th percentiles. Lines in the boxes show the median values. Data are the means of nine replicates: three growing seasons and three replications per treatment.

Figure 1 presents the effect of irrigation and fertilization treatments on the yield, WUE and PFP in three consecutive growing seasons in 2012−2013. The interactions between the irrigation and fertilization treatments and the individual factors of irrigation or fertilization significantly affected fruit yield, WUE and PFP (Supplementary Table S1). The irrigation and fertilization treatments significantly increased yields (Fig. 1A). The mean fruit yields of the F2 and F3 treatments were 3.57% and 11.37% lower, respectively, than the F1 yields in both years. The average fruit yields of the W2 and W3 treatments were 6.27% and 12.87% lower, respectively, than the W1 yields in both years. The highest yield was 95.8 tons ha^−1^ in the W1F1 treatment, which was significantly higher (6.15% to 25.69%) than the yields from the other treatments (Supplementary Table S2).

WUE was strongly related to the type of irrigation treatment used (Fig. 1B). The ranking of the irrigation treatments, from high to low average WUE, was W3 > W2 > W1. The mean WUE for the W3 treatment was 43.97 kg m^−3^, which was 19.36% and 28.51% higher than in the W2 and W1 treatments, respectively (Supplementary Table S2). However, a positive interaction was observed in the effect of the fertilization treatment on WUE. The mean WUE for the F1 treatment was 38.96 kg m^−3^, which was 3.13% and 12.33% higher than in the F2 and F3 treatments, respectively.

The highest PFP was 305.81 kg kg^−1^ in the W1F3 treatment. The mean PFP in the W1 treatment was 6.15% and 13.46% higher than in the W2 and W3 treatments, respectively. The influence of the fertilizer treatment on PFP was greater than the influence of the irrigation treatment (Supplementary Table S1); the average PFP in the F1 treatment was 26.41% and 42.43% lower than in the F2 and F3 treatments, respectively. For each irrigation level, PFP significantly decreased as the fertilization level increased from F3 to F2 to F1. In contrast, PFP increased with increases in the level of irrigation at each fertilization level (Fig. 1C).

### Response of fruit quality to irrigation and fertilization at different water and fertilizer input levels

**Figure 2:**
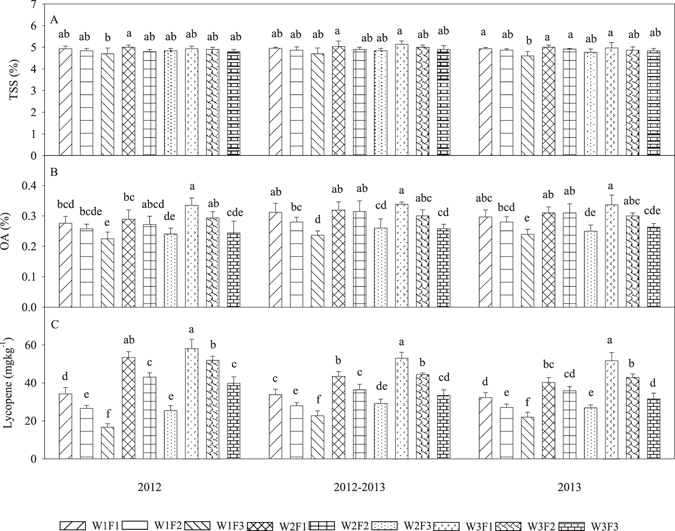
Effects of different irrigation and fertilization treatments on total soluble solids (TSSs) (**A**), the organic acid (OA) content (**B**) and the lycopene content (**C**) in fruit in three consecutive growing seasons in 2012−2013. Columns with the same letter represent values that are not significantly different at the 0.05 level of probability according to the LSD test. Each value is the mean ± SD (n = 3). The treatments are noted as W1, 100% *ET*_*0*_; W2, 75% *ET*_*0*_; W3, 50% *ET*_*0*_; F1, N240−P_2_O_5_120−K_2_O150 kg ha^−1^; F2, N180−P_2_O_5_90−K_2_O112.5 kg ha^−1^; and F3, N120−P_2_O_5_60−K_2_O75 kg ha^−1^.

The effects of irrigation and fertilization on the total soluble solid (TSS), organic acid (OA) and lycopene contents in the three consecutive growing seasons of the experiment are summarized in Fig. 2. The interactions between irrigation and fertilization were an important factor for the OA and lycopene contents, and the individual factor of irrigation or fertilizer was significantly related to the TSS, OA and lycopene contents; however, there was no significant interaction between irrigation and fertilization in relation to TSS content (Supplementary Table S3). TSS contents also increased with increasing fertilizer levels, but this effect was not significantly different between the treatment groups (Fig. 2A). The analysis indicates that a moderate water and high fertilizer (W2F1) input resulted in a relatively high TSS content in both years (Supplementary Fig. S1).

The highest OA content was obtained from the W3F1 treatment, and the F1 treatments had a significantly higher OA content than the F3 treatments in the three consecutive growing seasons (Fig. 2B). The mean OA content in the F1 treatment was 7.30% and 21.18% higher than in the F2 and F3 treatments, respectively. Conversely, the mean OA content increased with reductions in the water supply (Supplementary Fig. S2).

In the three consecutive growing seasons, the lycopene content was significantly higher (*p* < 0.05) in the W3F1 treatment than in the other treatments (Fig. 2C), showing that a higher fertilizer application rate results in a higher lycopene content. Under the same fertilization conditions, a higher lycopene content resulted from lower levels of irrigation (Supplementary Fig. S3).

**Figure 3:**
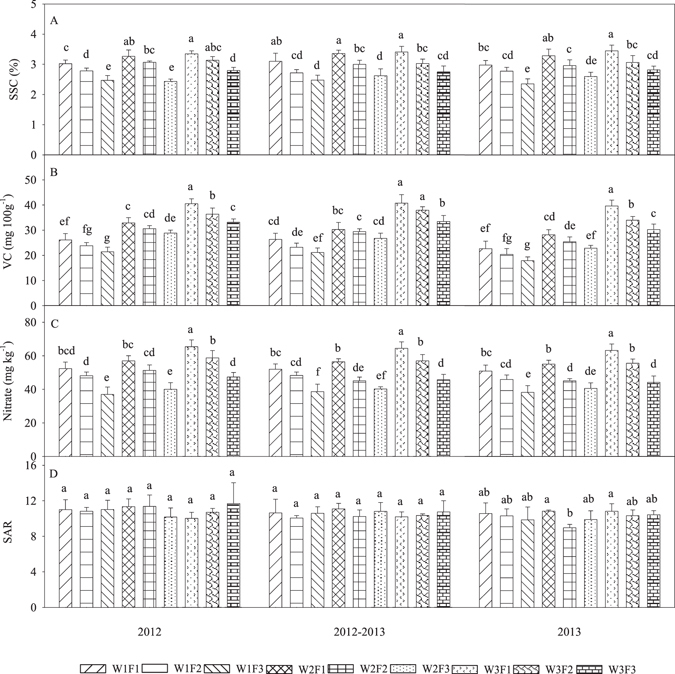
Effects of different irrigation and fertilization treatments on the soluble sugar content (SSC), vitamin C (VC) content, nitrate concentration (NC) and sugar/acid ratio (SAR) in fruit in three consecutive growing seasons in 2012−2013. Columns with the same letter represent values that are not significantly different at the 0.05 level of probability according to the LSD test. Each value is the mean ± SD (n = 3). The treatments are noted as W1, 100% *ET*_*0*_; W2, 75% *ET*_*0*_; W3, 50% *ET*_*0*_; F1, N240−P_2_O_5_120−K_2_O150 kg ha^−1^; F2, N180−P_2_O_5_90−K_2_O112.5 kg ha^−1^; and F3, N120−P_2_O_5_60−K_2_O75 kg ha^−1^.

The effects of irrigation and fertilization on the soluble sugar content (SSC), vitamin C (VC) content, nitrate concentration (NC) and the sugar/acid ratio (SAR) in the 2012, 2012−2013, and 2013 seasons are given in Fig. 3. The SSC in the irrigation and fertilization treatments in the three consecutive growing seasons ranged from 2.19% to 3.59% (Fig. 3A), with the lowest levels recorded in the W1F3 treatment and the highest levels recorded in the W3F1 treatment. A trend of increasing SSC with increasing rates of fertilizer application was observed (Supplementary Fig. S4). The individual factors of irrigation or fertilization significantly affected the SSC, but there was no significant interaction between irrigation and fertilization on the SSC (Supplementary Table S4).

The VC content in the tomatoes in the three consecutive growing seasons decreased significantly as the level of irrigation increased (Fig. 3B). Mean VC contents ranged from 20.16 to 40.31 mg 100 g^−1^. The mean VC content in the F1 treatment was 9.16%, 17.98% higher than in the F2 and F3 treatments, respectively. Conversely, the mean VC content in the W1 treatment was 2.57%, 6.06% lower than in the W2 and W3 treatments, respectively (Supplementary Fig. S5). The interactions between the irrigation and fertilizer treatments were important for the VC content, and the individual factors of irrigation or fertilizer were very significant (*p* < 0.01) in relation to the VC content in both years (Supplementary Table S4).

The NC values ranged from 32.80 to 70.13 mg kg^−1^ in the three consecutive growing seasons under different irrigation and fertilizer treatments (Fig. 3C). The highest mean NC was 64.3 mg kg^−1^ in the W3F1 treatment, which was significantly higher (from 11.29% to 41.03%) than in the other treatments (Supplementary Fig. S6). There was a significant effect of the interaction between irrigation and fertilization on the NC (Supplementary Table S5).

There was no significant difference between the irrigation and fertilization treatments in the SARs in the 2012 and 2012−2013 seasons (Fig. 3D). The W2F1 treatment resulted in a relatively high mean SAR (Supplementary Fig. S7). There was no significant effect of the interaction between irrigation and fertilization on SARs (Supplementary Table S5).

### Rankings of fruit quality based on a PCA

Based on all the collected data for the fruit quality parameters in three consecutive growing seasons (Supplementary Table S6), it is theoretically possible to consider the standardized values as variables representing fruit quality (Supplementary Table S7). A correlation matrix was calculated from the standardized values (Supplementary Table S8). Then, the total variance explained by the contribution rate (*C*_*r*_) and the accumulative contribution rate (*AC*_*r*_), based on eigenvalues, which was obtained by PCA (Supplementary Table S9). In this analysis, two components were extracted from the matrix of fruit quality parameters (Supplementary Table S10, Supplementary Fig. S8). The comprehensive quality rankings based on the PCA are shown in Table 1; W2F1 was ranked first, followed by W3F1, and W1F3 was clearly last.

**Table 1:** The score and rank of the comprehensive tomato quality parameters calculated in a PCA of all the treatments.

Treatment	Q_1_	Q_2_	Q	Rank
W1F1	0.305	0.708	1.014	4
W1F2	−1.335	−0.304	−1.639	7
W1F3	−3.796	0.010	−3.786	9
W2F1	2.158	1.687	3.844	1
W2F2	0.090	−1.195	−1.105	6
W2F3	−2.227	−0.659	−2.886	8
W3F1	3.784	−1.023	2.761	2
W3F2	1.662	−0.508	1.153	3
W3F3	−0.640	1.284	0.644	5

The treatments are noted as W1, 100% *ET*_*0*_; W2, 75% *ET*_*0*_; W3, 50% *ET*_*0*_; F1, 240N−120P_2_O_5_−150K_2_O kg ha^−1^; F2, 180N−90P_2_O_5_−112.5K_2_O kg ha^−1^; and F3, 120N−60P_2_O_5_−75K_2_O kg ha^−1^.

### Rankings of WUE, PFP, and fruit yield and quality based on a PCA

The means of the original WUE, PFP, and fruit yield and quality values were calculated for the three consecutive growing seasons in 2012−2013 (Supplementary Table S11). This value was then used in the following steps: (1) the data were converted to standardized values (Supplementary Table S12), (2) the correlation matrix was calculated using the standardized values (Supplementary Table S13), and (3) the total variance explained by *C*_*r*_ and *AC*_*r*_, based on eigenvalues, was obtained via a PCA (Supplementary Table S14). In this analysis, three components were extracted from the matrix of fruit quality parameters (Supplementary Table S15, Supplementary Fig. S9). The comprehensive quality ranks based on the PCA are shown in Table 2; W2F1 was the first, followed by W1F1, and W1F3 was clearly last.

**Table 2:** The score and rank of the comprehensive fruit yield and quality, WUE, and PFP values calculated in a PCA of all the treatments.

Treatment	Q_1_	Q_2_	Q_3_	Q	Rank
W1F1	0.448	2.220	0.321	2.990	2
W1F2	−1.435	1.335	−0.570	−0.670	5
W1F3	−4.337	−0.185	0.025	−4.498	9
W2F1	2.294	1.152	1.506	4.951	1
W2F2	0.103	0.166	−1.262	−0.993	7
W2F3	−2.622	−0.855	−0.529	−4.006	8
W3F1	4.208	−0.811	−0.870	2.527	3
W3F2	2.027	−1.259	−0.267	0.501	4
W3F3	−1.762	−0.686	1.646	−0.801	6

The treatments are noted as W1, 100% *ET*_*0*_; W2, 75% *ET*_*0*_; W3, 50% *ET*_*0*_; F1, 240N−120P_2_O_5_−150K_2_O kg ha^−1^; F2, 180N−90P_2_O_5_−112.5K_2_O kg ha^−1^; and F3, 120N−60P_2_O_5_−75K_2_O kg ha^−1^.

## Discussion

A systematic and quantitative analysis of the effects of irrigation and fertilization levels on tomato fruit yield and quality, WUE, and PFP was performed using solar greenhouse experiments in three consecutive seasons. We focused on tomatoes because of their global popularity as a vegetable and their characteristics as a healthy food that is rich in vitamins, OAs, lycopene, antioxidants and essential amino acids^26–29^. During the analysis, we emphasized fruit quality because of increasing consumer demand for high quality food with recent rapid economic developments^30, 31^. The results of our study indicate that the interactions between irrigation and fertilization significantly affect the yield, WUE, PFP and quality of tomatoes during the growing season.

Fruit yield and WUE were more sensitive to irrigation than to fertilization, and PFP was more sensitive to fertilization than to irrigation. Both irrigation and fertilization are essential factors for tomato growth, and they both influence fruit yield and quality^17, 32, 33^. An inadequate irrigation rate could decrease tomato yields to some extent while improving fruit quality^34–36^. Fertilization is important for tomato growth, and the proper amount of fertilizer actively promotes improved tomato fruit quality^24^. In this study, the highest yield was 95.8 tons ha^−1^ in the W1F1 treatment, which improved the fruit yield from 6.15% to 25.69% relative to the yields from the other treatments. In addition, fruit size was significantly smaller when irrigation was stopped early, and a greater water deficit during the beginning of the vegetative growth stage also reduced yield. The highest fruit yield was obtained in the I_100_ × N_180_ (I_100_, 100% ET_c_; N_180_, 180 kg nitrogen fertilizer ha^−1^) irrigation treatment due to the sufficient level of irrigation and optimal fertigation, which also resulted in the highest fruit yield^4, 35, 37, 38^. Tomatoes often grow during the fruit enlargement period when their water and nutrient demands are high mainly because many small green fruits are well maintained or do not enlarge under poor conditions with a water shortage. In solar greenhouses, for example, the yield can more than double under full irrigation compared to yields under very stressful conditions^34, 39^.

The effects of irrigation and fertilization on WUE and PFP were evaluated at different water and fertilizer input levels. WUE and PFP decreased significantly as the irrigation and fertilization levels increased over three consecutive seasons. However, WUE increased rapidly as the irrigation level decreased, until it reached 45.3 kg m^−3^, which was similar to the change in PFP, which reached 305.8 kg kg^−1^ when the fertilization input level decreased. A high level of fertilization also improved the fruit yield in the irrigated treatment with 75% *ET*_*0*_ (W2F1), whereas the same level of irrigation had no or very little influence on fruit yields at lower levels of fertilization. The individual factors of irrigation or fertilization and the interactions between the irrigation and fertilization treatments were very significant (*p* < 0.001) for fruit yield, WUE and PFP. We can safely conclude that irrigation and fertilization act synergistically in their effects on fruit yield, WUE and PFP, and therefore all must be analysed.

The TSS and OA contents were more sensitive to fertilization than to irrigation, but the changes in the VC content showed an opposite effect. The interactions between the irrigation and fertilization treatments were significant (*p* < 0.05) for OAs, lycopene, VC and NC. Lycopene and VC are two important antioxidants and represent the main nutritional qualities of tomatoes^40, 41^. In the three consecutive years, lycopene and VC contents were significantly higher (*p* < 0.05) in the W2F1 and W3F1 treatments than in the other treatments (Figs 2 and 3), indicating that fruit quality was improved by high fertilizer levels and a low irrigation rate. These results appear to contradict the assumptions that greater water and fertilizer inputs increase fruit yields. Therefore, we should strive for a balance between the supply and demand of water and fertilizer in modern management technology in solar greenhouse grown tomatoes. As mentioned above, with rapid economic development, consumer demands for higher quality food are increasing. Thus, we should not concentrate on only maximizing fruit yield to the neglect of fruit quality. In general, fruit yield and fruit quality cannot simultaneously reach their maximum values. In this study, we used PCA methods to evaluate a comprehensive index of tomato production (fruit yield, fruit quality, WUE, PFP), and we identified the optimal combination of irrigation and fertilization, which is the aim of agricultural water and fertilizer management.

Moderate irrigation and high fertilizer levels (W2F1) resulted in the highest quality index scores relative to the other treatments. The fruit yield of the W2F1 treatment was 10.54% lower than that of the W1F1 treatment, and the WUE of the W2F1 treatment was 7.25% higher than that of the W1F1 treatment. Among the antioxidant compounds in tomatoes, lycopene and VC are the most important^42–45^. In particular, the lycopene and VC contents were significantly higher in the W2F1 treatment than in the W1F1 treatment. The overall scores for an individual quality, yield, WUE or PFP attribute is determined by the number of individual quality, yield, WUE and PFP attributes that are included. For example, although yield was the criterion with the greatest weight, the overall weight of the WUE, PFP, TSS, SSC, lycopene, VC and OA attributes decreased the influence of yield when all eight single-factor attributes were included. The comprehensive yield, WUE, PFP and quality index ranks should be as consistent with the single-factor performance ranks as possible.

Full irrigation (100% *ET*_*0*_) and a high fertilizer level (N240−P_2_O_5_120−K_2_O150 kg ha^−1^) maximized the fruit yield from solar greenhouse tomatoes when compared with plants cultivated at different water and fertilizer input levels. However, a 75% *ET*_*0*_ irrigation strategy could be adopted, especially in areas where water resources are increasingly scarce, such as northwest China. In addition, the TSS, OA, lycopene, SSC, VC, NC and SAR values in the W2F1 treatment were 1.44%, 3.70%, 26.87%, 8.15%, 17.68%, 7.81% and 3.13% higher, respectively, than in the W1F1 treatment. Meanwhile, the W2F1 treatment resulted in a yield reduction that was less than proportional to the water savings, and it improved fruit quality. Moreover, the significant positive correlation coefficient between irrigation and fertilization shows that the rankings of the W2F1 treatment remained stable when the PCA method was used to calculate the ranks of the individual fruit-quality parameters and when fruit yield and quality, WUE and PFP were used. Indeed, previous studies have suggested that irrigation should be applied during the whole growing season, even at a lower rate to reach satisfactory yields^38, 46^.

The results of the present study indicate that the W2F1 treatment ranked first in both analyses (Tables 1 and 2) after the combinational PCA evaluation. The analysis showed an obvious trade-off among the fruit yield, WUE, PFP and fruit quality of the tomatoes. Hence, the W2F1 treatment (75% *ET*_*0*_, N240−P_2_O_5_120−K_2_O150 kg ha^−1^) represents the best water and fertilizer management plan for greenhouse tomato crops in northwest China.

## Materials and Methods

### Plant material and culture

**Figure 4:**
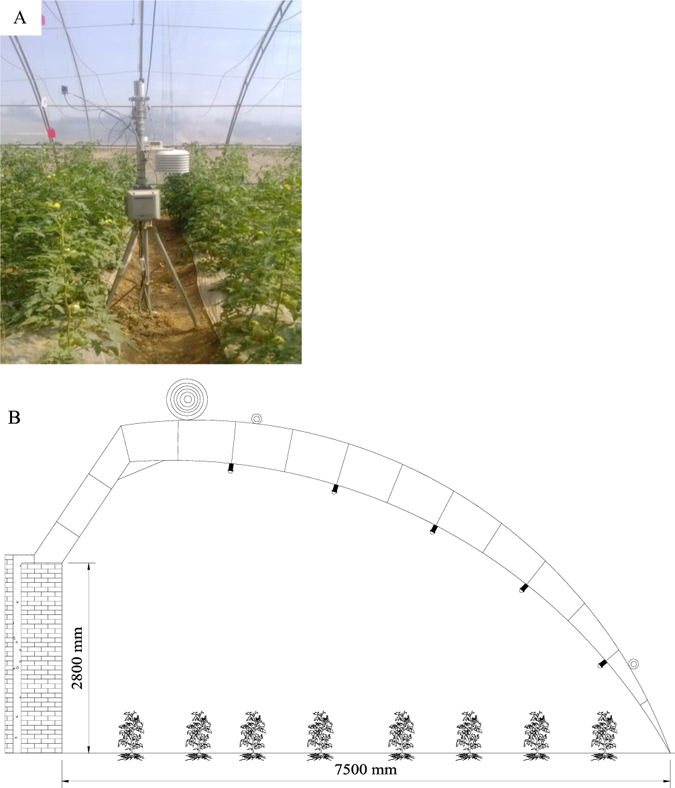
Photograph of the automatic weather station located in the greenhouses (**A**), and a schematic diagram of the arrangement of the greenhouse system (**B**).

Tomato (*Solanum lycopersicum Mill*., cv. ‘Jinpeng 10’) plants were grown in the greenhouse of the Key Laboratory of Agricultural Soil and Water Conservation Engineering in Arid Areas (34°20′N, 108°04′E and altitude 521 m), Yang Ling County, Shaanxi Province, China. The atmospheric pressure, temperature, light and photosynthetically active radiation (PAR), relative humidity and solar radiation inside the greenhouse were recorded using an automatic weather station (HOBO event logger, USA), which was located in the centre of the greenhouse (Fig. 4A). The greenhouse was oriented east-west, with an area of 570 m^2^ (7.5 m in width and 76 m in length) at a height of 2.8 m (Fig. 4B). The soil in the solar greenhouse had a heavy loam texture, according to the USDA texture classification system, and was derived from loess with a deep and even soil profile. Two rows of tomato plants were transplanted on the bed top on 21-Mar, 3-Sep 2012 and 31-Mar 2013. Furrow-film mulch was cultivated using the local traditional planting patterns and calendars using tomato ridging in a tube with a two-line layout, spaced 50 cm apart, with a 45-cm planting distance and 78 plants in each experimental plot.

### Experimental design and drip irrigation system

In this experiment, nine treatments were designed with three different irrigation levels (W1: 100% *ET*_*0*_; W2: 75% *ET*_*0*_; W3: 50% *ET*_*0*_) and fertilizer levels (F1: 240N−120P_2_O_5_−150K_2_O kg ha^−1^; F2: 180N−90P_2_O_5_−112.5K_2_O kg ha^−1^; F3: 120N−60P_2_O_5_−75K_2_O kg ha^−1^). The experiment was organized using a randomized block design with three replicates; each plot was 6 m long and 3.75 m wide (22.5 m^2^) for each treatment (6 m × 3.75 m = 22.5 m^2^). There were nine ridged experimental plots, which were divided by a water barrier sheet.

**Figure 5:**
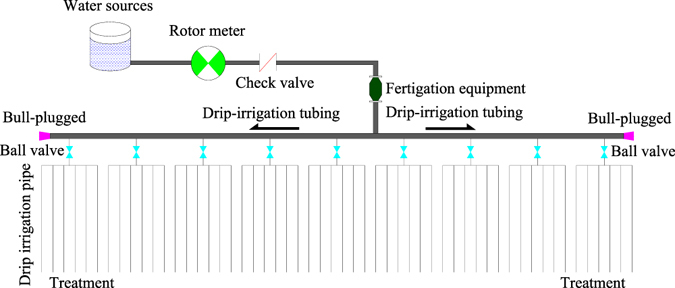
A drip irrigation system consisting of a rotor metre, check valve, drip fertigation equipment, drip irrigation tubing, ball valve, and drip irrigation pipe.

The drip line consisted of an inserted cylinder head drip irrigation pipe with an inner diameter of 8 mm, a drop head span of 30 cm, a head flow of 2 L h^−1^, and a drip irrigation operating pressure of 0.3 MPa (Fig. 5).

### Irrigation

The irrigation treatments were initiated using the surface drip irrigation system during transplanting, and 40 mm of water was provided. The irrigation treatments were based on the sum of reference crop evapotranspiration (*ET*_*0*_) rates between two adjacent irrigation times. The FAO 56 Penman-Monteith method, recommended as the standard method for estimating *ET*_*0*_, was used^47^. Fernández *et al*.^48^ reported that the following FAO 56 Penman-Monteith equation with a fixed aerodynamic resistance of 295 s m^−1^ can better estimate daily *ET*_*0*_ in a greenhouse:

1 $$E{T}_{0}=\tfrac{0.48{\rm{\Delta }}({R}_{n}-G)+\gamma (628/(T+273))({e}_{s}-{e}_{a})}{{\rm{\Delta }}+628\gamma }$$

where *R*_*n*_ is the net radiation (MJ m^−2 ^d^−1^), *G* is the soil heat flux (MJ m^−2 ^d^−1^), *Δ* is the slope of the saturated vapour pressure curve (kPa °C^−1^), *γ* is the psychometric constant (kPa °C^−1^), *e*_*s*_ is the saturation vapour pressure (kPa), *e*_*a*_ is the actual vapour pressure (kPa), and *e*_*s*_ − *e*_*a*_ (*VPD*) is the vapour pressure deficit (kPa). The calculation procedures for the parameters *R*_*n*_, *G*, *e*_*s*_, *e*_*a*_, *Δ*, *γ* and *T* are described in the FAO 56 guidelines^47, 49–51^. The average daily environmental conditions at different growth stages of the tomatoes inside the greenhouse, the seasonal variation of the daily *ET*_*0*_ calculated using equation (1), and the sum of *ET*_*0*_ in different growth stages are shown in Table 3.

**Table 3:** Average daily environmental parameters recorded at different growth stages of tomatoes inside the greenhouse.

Season	Growth stage	Rs (W m^−2^)	Ta (°C)	RH (%)	VPD (kPa)	ET_0_ (mm)	Irrigation (mm)			Fertilization (Kg ha^−1^)		
							W1	W2	W3	F1	F2	F3
2012	Recovering stage (21-Mar–11-Apr)	99.35	15.86	73.59	0.75	48.40	40.00	40.00	40.00	63.75	47.81	31.88
	Blossoming and bearing fruits stage (12-Apr–23-Apr)	115.69	21.70	74.09	0.89	35.60	35.60	26.70	17.80	63.75	47.81	31.88
	First fruit enlargement period (24-Apr–15-May)	135.68	21.44	82.71	1.06	47.20	47.20	35.40	23.60	127.50	95.63	63.75
	Second fruit enlargement period (16-May–28-May)	132.32	21.58	80.57	1.22	31.90	31.90	23.93	15.95	127.50	95.63	63.75
	Third fruit enlargement period (29-May–7-Jul)	163.51	24.86	74.49	1.51	107.30	107.30	80.48	53.65	127.50	95.63	63.75
	Entire season	136.30	21.67	76.68	1.17	270.40	262.00	206.50	151.00	510.00	382.50	255.00
2012–2013	Recovering stage (3-Sep–26-Sep)	120.40	19.32	72.35	1.03	54.60	40.00	40.00	40.00	63.75	47.81	31.88
	Blossoming and bearing fruits stage (27-Sep–12-Oct)	106.57	18.25	81.26	0.61	25.92	25.92	19.44	12.96	63.75	47.81	31.88
	First fruit enlargement period (13-Oct–31-Oct)	90.27	17.55	75.36	0.48	23.76	23.76	17.82	11.88	127.50	95.63	63.75
	Second fruit enlargement period (1-Nov–22-Nov)	78.65	16.65	84.01	0.40	28.60	28.60	21.45	14.30	127.50	95.63	63.75
	Third fruit enlargement period (23-Nov–12-Feb)	74.85	15.98	78.88	0.44	104.53	104.53	78.40	52.27	127.50	95.63	63.75
	Entire season	89.83	17.20	78.35	0.57	237.41	222.81	177.11	131.41	510.00	382.50	255.00
2013	Recovering stage (31-Mar–15-Apr)	85.29	17.42	68.74	0.68	30.30	40.00	40.00	40.00	63.75	47.81	31.88
	Blossoming and bearing fruits stage (16-Apr–25-Apr)	108.95	19.26	72.69	0.85	27.10	27.10	20.33	13.55	63.75	47.81	31.88
	First fruit enlargement period (26-Apr–14-May)	129.65	22.39	76.41	0.95	48.70	48.70	36.53	24.35	127.50	95.63	63.75
	Second fruit enlargement period (15-May–30-May)	133.58	22.33	83.79	1.18	45.50	45.50	34.13	22.75	127.50	95.63	63.75
	Third fruit enlargement period (31-May–15-Jul)	158.68	26.21	76.21	1.42	118.50	118.50	88.88	59.25	127.50	95.63	63.75
	Entire season	134.37	23.00	76.07	1.14	270.10	279.80	219.85	159.90	510.00	382.50	255.00

*R*_*s*_ is the solar radiation, *T*_*a*_ is the air temperature, RH is the relative humidity, *VPD* is the vapour pressure deficit, *ET*_*0*_ is the reference evapotranspiration; irrigation treatments (W1, W2 and W3); fertilizer treatments (F1, F2 and F3).

### Fertilizer

A hydraulic proportional pump was used to determine precisely how much fertilizer to apply. Drip fertigation was performed with a fertilizer composed of urea (46% N), diammonium phosphate (44% P_2_O_5_) and potassium chloride (60% K_2_O) that was applied five times (at the recovering stage, blossoming and fruit-bearing stage, first fruit enlargement period, second fruit enlargement period and third fruit enlargement period) at a fertilization ratio of 1:1:2:2:2. The water metre and hydraulic proportional fertilization pump accurately controlled the irrigation water and fertilizer application. Drip irrigation fertilization treatments were performed during the reproductive period for water irrigation and fertilization (Table 3).

### Soil Water Balance

Crop evapotranspiration (*ET*_*c*_) was calculated using the water balance method by monitoring the change in the soil water content over a period of time. *ET*_*c*_ was estimated using the following water balance equation^52^:

2 $$E{T}_{c}=I-\Delta S-R-D$$

where *ET*_*c*_ is the crop water consumption (mm); *I* is the amount of irrigation water (mm); *ΔS* is the change in soil water storage (mm) before sowing and after harvest, which was measured gravimetrically; the depth interval spacing was 10 cm; *R* is the run-off (mm); and *D* is the drainage (mm). There was no precipitation in the greenhouse and no surface runoff from the plots because all furrows were blocked and the surface runoff was negligible; therefore, *R* = 0. Non-weighting, percolation-type lysimeters (1-mm-thick steel) that were 1.0 m long and 1.2 m wide were installed 0.6 m beneath the bottom of the furrow to collect drainage water. However, no drainage was observed from the lysimeters in each of the plots; thus, *D* = 0. The overall soil water balance in two consecutive years is shown in Table 3.

### Measurement of fruit yield, WUE and fertilizer PFP

Ripe tomatoes were harvested, and the total fresh tomato yield from all plants in each plot was measured at each harvesting. The fruit yield was measured throughout the crop. The fruits were harvested twice a week for a period of nine weeks and determined based on electronic weighing.

The WUE (in kg m^−3^) was determined using the following equation^2, 53^:

3 $$WUE=Y/E{T}_{c}\times 0.1$$

where *Y* is the total fruit yield (kg ha^−1^), and *ET*_*c*_ is the crop water consumption (mm).

The PFP of the fertilizer (in kg kg^−1^) was determined using the following equation^2, 54–57^.

4 $$PFP=Y/F$$

where *Y* is the total fruit yield (kg ha^−1^), and *F* is the fertilizer applied (kg ha^−1^), i.e., the sum of nitrogen, phosphate and potassium fertilizer applied during each crop-growing season.

### Measurement of fruit quality

The fruit quality was measured during the third fruit enlargement period. For each measurement, five fruits of similar size and maturity and with no external defects were chosen from each plot. The vitamin C (VC) content was measured using the molybdenum blue colorimetric method^58^, and the anthrone and sulfuric acid colorimetric methods were used to determine the soluble sugar content (SSC)^59, 60^. The total soluble solid (TSS) content was measured using a digital refractometer (Link Co. Ltd., Taiwan, China), the nitrate concentration (NC) was measured using ultraviolet-spectrophotometry, lycopene was measured using ultraviolet-visible light spectrophotometry^61^, and the amount of organic acid (OA) was determined via titration with NaOH (0.1 mol L^−1^)^62^. The sugar-acid ratio (SAR) was calculated as the SSC divided by the OA content.

### Statistical analysis

An analysis of variance was conducted on the fruit yield, WUE, PFP, SSC, TSS, NC, lycopene, VC, OA and SAR using a two−way analysis of variance (GLM procedure in SAS version 9.2, SAS Institute Ltd., North Carolina, USA). Tukey’s HSD multiple range test results were considered significant at *p* < 0.05.

### PCA of the yield, WUE, PFP and fruit quality of greenhouse tomatoes

PCA is the general name for a technique that uses sophisticated underlying mathematical principles to transform a number of possibly correlated variables into a smaller number of variables called principal components^63–65^. The process for the analysis is as follows:

I) Select sample parameters. Normalization seeks to obtain comparable scales, which allow for attribute comparisons. The dimensionality reduction approach involves minimizing the squared errors via a vector coordinate transformation, and the measurement data are defined based on the following equation:

5 $$X=[\begin{array}{cccc}{X}_{11} & {X}_{12} & \cdots  & {X}_{1p}\\ {X}_{21} & {X}_{22} & \cdots  & {X}_{2p}\\ \vdots  & \vdots  &  & \vdots \\ {X}_{n1} & {X}_{n2} & \cdots  & {X}_{np}\end{array}]$$

where *n* is the measured value of the sample number (i.e., yield of tomato fruit, WUE, PFP and fruit quality in this study), and *p* is the variable number.

II) Sample parameters are converted to standardized values. It is convenient to standardize the sample with the following equation:

6 $${x}_{ij}^{\ast }=\frac{{x}_{ij}-{\bar{x}}_{j}}{{s}_{j}}\quad \quad i=1,2,\ldots ,n;j=1,2,\ldots ,p$$

where ${\bar{x}}_{j}=\frac{1}{n}\sum _{i=1}^{n}{x}_{ij}$, ${s}_{j}^{2}=\frac{1}{n-1}\sum _{i=1}^{n}{({x}_{ij}-{\bar{x}}_{j})}^{2}$, and *n* is the measured value of the sample number.

III) The correlation matrix is calculated for the different irrigation and fertilization treatments and is defined based on the following equation:

7 $$R=[\begin{array}{cccc}{r}_{11} & {r}_{12} & \cdots  & {r}_{1p}\\ {r}_{21} & {r}_{22} & \cdots  & {r}_{2p}\\ \vdots  & \vdots  &  & \vdots \\ {r}_{p1} & {r}_{p2} & \cdots  & {r}_{pp}\end{array}]$$

where *r*_*ij*_ is the correlation coefficient of the original variable, *r*_*ij*_ = *r*_*ji*_, and *r*_*ij*_ is given by the following equation:

8 $${r}_{ij}=\frac{\sum _{k=1}^{n}({X}_{ki}-{\bar{X}}_{i})({X}_{kj}-{\bar{X}}_{j})}{\sqrt{\sum _{k=1}^{n}{({X}_{ki}-{\bar{X}}_{i})}^{2}\sum _{k=1}^{n}{({X}_{kj}-{\bar{X}}_{j})}^{2}}}$$

IV) The eigenvalues of the *R* values and the eigenvectors for each sample number are calculated. A Jacobi iteration is used to determine the eigenvalues, as defined in the following equation:

9 $$|\lambda E-R|=0$$

where *λ* is the eigenvalue, E is the identity matrix and *R* is the correlation matrix. Next, these eigenvalues are sized down as *λ*_1_ ≥ *λ*_2_ ≥ … ≥*λ*_*p*_ ≥ 0, and the respective eigenvector *e*_*i*_ (*i* = 1, 2, ……) solved for:

$$\Vert {e}_{i}\Vert =1\quad \sum _{j=1}^{p}{e}_{ij}^{2}=1$$

where *e*_*ij*_ is the *j-*th component of *e*_*i*_.

V) The contribution rate (*C*_*r*_) and accumulative contribution rate (*AC*_*r*_), with eigenvalues, are calculated using the following equations:

10 $${C}_{r}=\frac{{\lambda }_{i}}{\sum _{k=1}^{p}{\lambda }_{k}}\,(i=1,2,\cdots ,p)$$

11 $$A{C}_{r}=\frac{\sum _{k=1}^{i}{\lambda }_{k}}{\sum _{k=1}^{p}{\lambda }_{k}}\,(i=1,2,\cdots ,p)$$

From the calculation results, the principal components corresponding to the characteristic value were greater than 1. The sample number of the principal components was selected as *t*; then, the factor of the former *t* was used as the corresponding data object associated with the component matrix *S*_*1*_, *S*_*2*_, *···*, *S*_*t*_.

VI) The mathematical model is established based on the PCA, as defined in the following equation:

12 $$\begin{array}{ccc}{Q}_{1} & = & {S}_{11}{X}_{1}+{S}_{12}{X}_{2}+\ldots +{S}_{1p}{X}_{p}\\ {Q}_{2} & = & {S}_{21}{X}_{1}+{S}_{22}{X}_{2}+\ldots +{S}_{2p}{X}_{p}\\  &  & \cdot \cdot \cdot \cdot \cdot \cdot \\ {Q}_{t} & = & {S}_{t1}{X}_{1}+{S}_{t2}{X}_{2}+\ldots +{S}_{tp}{X}_{p}\end{array}$$

where *S*_*1i*_, *S*_*2i*_, …, *S*_*ti*_ (*i *=* 1*, *2*, …, *t*) are the eigenvectors corresponding to the principal components, and *X*_*1*_, *X*_*2*_, …, *X*_*p*_ are the standardized values, the value of which is converted based on the sample parameters.

VII) The evaluation process is determined based on the comprehensive evaluation index (F). The F value is defined by the following equation:

13 $$Q={\lambda }_{1}{Q}_{1}+{\lambda }_{2}{Q}_{2}+\ldots +{\lambda }_{t}{Q}_{t}$$

where *λ*_1_, *λ*_2_ … *λ*_*t*_ are the characteristic values corresponding to the principal components, and *Q*_1_, *Q*_2_, …, *Q*_*t*_ are the evaluation values of the different irrigation and fertilization treatments. A higher comprehensive evaluation index indicates a better treatment.

## Conclusions

A scientific irrigation schedule should involve a compromise that comprehensively considers the effects of water stress on not only the yield and fruit quality but also the water conservation capacity. Similarly, an optimal fertilizer application level should aim to improve fruit yield and quality, as well as minimize fertilizer waste. The synergism and interaction of irrigation and fertilization are very important for solar greenhouse crop water and fertilizer management. The proper application of drip fertigation (W2F1) may help to obtain a good compromise among the yield, WUE, PFP and fruit quality of solar greenhouse tomatoes, improving fruit quality and saving large amounts of water. This is particularly important in arid and semiarid areas, such as that of the present experiment, where water scarcity is an increasing concern and water costs are continuously rising. In addition, the tomatoes produced in the W2F1 treatment could be of great benefit to human health because of their higher OA, lycopene and VC contents.

Regardless of the research achievements and promotional activities regarding greenhouse management, traditional methods of frequent watering and heavy fertilizer use remain common practice, and considerable efforts will have to be made to achieve the widespread application of water and fertilizer conservation. The W2F1 treatment is recommended because it increased the WUE and lycopene and VC contents by 7.25%, 26.87% and 17.68%, respectively, compared with the W1F1 (irrigation, 100% *ET*_*0*_; fertilization, N240−P_2_O_5_120−K_2_O150 kg ha^−1^) treatment. Moreover, the W2F1 treatment ranked first after both analyses in the combinational evaluation: first when the PCA methods were used to comprehensively evaluate tomato fruit quality to determine the best water and fertilizer treatment and second when the comprehensive analysis included the tomato fruit yield and quality, WUE and PFP.

The present method sheds light on the contributions of these practices, clarifies their impacts, and provides a basis on which to evaluate and select better management practices in greenhouses. These results have major implications for improving the management of water and fertilizer inputs to solar greenhouse crops and implementing reasonable drip fertigation systems in northwest China and other parts of the world. Additionally, the optimization result from this study can be scheduled before sowing. In the future, we will focus on the levels of irrigation and fertilization, including more graded levels to better estimate the input rate of the W2F1 treatment.

## Electronic supplementary material

Supplementary Information

## References

[1] Bernacchi CJ, VanLoocke A (2015). Terrestrial ecosystems in a changing environment: a dominant role for water. Annu. Rev. Plant. Biol..

[2] Qin W, Wang D, Guo X, Yang T, Oenema O (2015). Productivity and sustainability of rainfed wheat-soybean system in the North China Plain: results from a long-term experiment and crop modelling. Sci. Rep.

[3] He Y, Hou L, Wang H, Hu K, McConkey B (2014). A modelling approach to evaluate the long-term effect of soil texture on spring wheat productivity under a rain-fed condition. Sci. Rep.

[4] Patanè C, Tringali S, Sortino O (2011). Effects of deficit irrigation on biomass, yield, water productivity and fruit quality of processing tomato under semi-arid Mediterranean climate conditions. Sci. Hortic.

[5] Di Paolo E, Rinaldi M (2008). Yield response of corn to irrigation and nitrogen fertilization in a Mediterranean environment. Field Crops Res.

[6] Fereres E, Soriano MA (2007). Deficit irrigation for reducing agricultural water use. J. Exp. Bot..

[7] Karam F (2007). Evapotranspiration, seed yield and water use efficiency of drip irrigated sunflower under full and deficit irrigation conditions. Agricult. Water Manage.

[8] Machado RMA, Rosário Md, Oliveira G, Portas CAM (2003). Tomato root distribution, yield and fruit quality under subsurface drip irrigation. Plant Soil.

[9] LeBauer DS, Treseder KK (2008). Nitrogen limitation of net primary productivity in terrestrial ecosystems is globally distributed. Ecology.

[10] Bar-Yosef B (1977). Trickle irrigation and fertilization of tomatoes in sand dunes: water, N, and P distributions in the soil and uptake by plants. Agron. J.

[11] Bar-Yosef B, Sagiv B (1982). Response of tomatoes to N and water applied via a trickle irrigation system. I. Nitrogen. Agron. J..

[12] Badr MA, Abou Hussein SD, El-Tohamy WA, Gruda N (2010). Nutrient uptake and yield of tomato under various methods of fertilizer application and levels of fertigation in arid lands. Gesunde Pflanz.

[13] Kumar S, Dey P (2011). Effects of different mulches and irrigation methods on root growth, nutrient uptake, water-use efficiency and yield of strawberry. Sci. Hortic.

[14] Tilman D, Cassman KG, Matson PA, Naylor R, Polasky S (2002). Agricultural sustainability and intensive production practices. Nature.

[15] Xu G, Fan X, Miller AJ (2012). Plant nitrogen assimilation and use efficiency. Annu. Rev. Plant. Biol..

[16] Robertson GP, Vitousek PM (2009). Nitrogen in agriculture: balancing the cost of an essential resource. Annu. Rev. Environ. Resour..

[17] Ozbahce A, Tari AF (2010). Effects of different emitter space and water stress on yield and quality of processing tomato under semi-arid climate conditions. Agricult. Water Manage.

[18] Pirmoradian N, Sepaskhah AR, Maftoun M (2004). Effects of water-saving irrigation and nitrogen fertilization on yield and yield components of rice (Oryza sativa L.). Plant Prod. Sci..

[19] Cakir R (2004). Effect of water stress at different development stages on vegetative and reproductive growth of corn. Field Crops Res.

[20] Jassal RS (2010). Impact of nitrogen fertilization on carbon and water balances in a chronosequence of three Douglas-fir stands in the Pacific Northwest. Agric. For. Meteorol.

[21] Hebbar SS, Ramachandrappa BK, Nanjappa HV, Prabhakar M (2004). Studies on NPK drip fertigation in field grown tomato (Lycopersicon esculentum Mill.). Eur. J. Agron..

[22] Singandhupe RB, Rao GGSN, Patil NG, Brahmanand PS (2003). Fertigation studies and irrigation scheduling in drip irrigation system in tomato crop (Lycopersicon esculentum L.). Eur. J. Agron..

[23] Zotarelli L, Dukes MD, Scholberg JMS, Muñoz-Carpena R, Icerman J (2009). Tomato nitrogen accumulation and fertilizer use efficiency on a sandy soil, as affected by nitrogen rate and irrigation scheduling. Agricult. Water Manage.

[24] De Pascale S, Maggio A, Orsini F, Barbieri G (2016). Cultivar, soil type, nitrogen source and irrigation regime as quality determinants of organically grown tomatoes. Sci. Hortic.

[25] Wang X, Li Z, Xing Y (2015). Effects of mulching and nitrogen on soil temperature, water content, nitrate-N content and maize yield in the Loess Plateau of China. Agricult. Water Manage.

[26] Erba D (2013). Nutritional value of tomatoes (Solanum lycopersicum L.) grown in greenhouse by different agronomic techniques. J. Food Compost. Anal.

[27] Toor RK, Savage GP, Heeb A (2006). Influence of different types of fertilisers on the major antioxidant components of tomatoes. J. Food Compost. Anal.

[28] Barański M (2014). Higher antioxidant and lower cadmium concentrations and lower incidence of pesticide residues in organically grown crops: a systematic literature review and meta-analyses. Br. J. Nutr..

[29] Masko EM, Allott EH, Freedland SJ (2013). The relationship between nutrition and prostate cancer: is more always better?. Eur. Urol..

[30] Tripathi A, Tripathi DK, Chauhan DK, Kumar N, Singh GS (2016). Paradigms of climate change impacts on some major food sources of the world: a review on current knowledge and future prospects. Agric. Ecosyst. Environ.

[31] Godfray HC (2010). Food security: the challenge of feeding 9 billion people. Science.

[32] Sun Y (2013). Simulating the fate of nitrogen and optimizing water and nitrogen management of greenhouse tomato in North China using the EU-Rotate_N model. Agricult. Water Manage.

[33] Topcu S (2007). Yield response and N-fertiliser recovery of tomato grown under deficit irrigation. Eur. J. Agron..

[34] Patanè C, Cosentino SL (2010). Effects of soil water deficit on yield and quality of processing tomato under a Mediterranean climate. Agricult. Water Manage.

[35] Marouelli WA, Silva WLC (2007). Water tension thresholds for processing tomatoes under drip irrigation in Central Brazil. Irrigation Sci.

[36] Du T, Kang S, Zhang J, Davies WJ (2015). Deficit irrigation and sustainable water-resource strategies in agriculture for China’s food security. J. Exp. Bot..

[37] Favati F (2009). Processing tomato quality as affected by irrigation scheduling. Sci. Hortic.

[38] Patanè C, Saita A (2015). Biomass, fruit yield, water productivity and quality response of processing tomato to plant density and deficit irrigation under a semi-arid Mediterranean climate. Crop Pasture Sci.

[39] Vaccari FP (2015). Biochar stimulates plant growth but not fruit yield of processing tomato in a fertile soil. Agric. Ecosyst. Environ.

[40] Dumas Y, Dadomo M, Lucca GD, Grolier P (2003). Effects of environmental factors and agricultural techniques on antioxidantcontent of tomatoes. J. Sci. Food Agric.

[41] Vallverdú-Queralt A (2012). Effects of pulsed electric fields on the bioactive compound content and antioxidant capacity of tomato fruit. J. Agric. Food Chem..

[42] Rao AV, Waseem Z, Agarwal S (1998). Lycopene content of tomatoes and tomato products and their contribution to dietary lycopene. Food Res. Int..

[43] Sun L (2012). Fruit-specific RNAi-mediated suppression of SlNCED1 increases both lycopene and β-carotene contents in tomato fruit. J. Exp. Bot..

[44] Giovannucci E (1999). Tomatoes, tomato-based products, lycopene, and cancer: review of the epidemiologic literature. J. Natl. Cancer Inst..

[45] Edward G, Rimm EB, Yan L, Stampfer MJ, Willett WC (2002). A prospective study of tomato products, lycopene, and prostate cancer risk. J. Natl. Cancer Inst..

[46] Shao GC, Deng S, Liu N, Wang MH, She DL (2015). Fruit quality and yield of tomato as influenced by rain shelters and deficit irrigation. J. Agric. Sci. Technol.

[47] Qiu R (2013). Response of evapotranspiration and yield to planting density of solar greenhouse grown tomato in northwest China. Agricult. Water Manage.

[48] Fernández MD (2010). Measurement and estimation of plastic greenhouse reference evapotranspiration in a Mediterranean climate. Irrigation Sci.

[49] Allen, R. G., Pereira, L. S., Raes, D. & Smith, M. Crop evapotranspiration-Guidelines for computing crop water requirements-FAO Irrigation and drainage paper 56. FAO, Rome **300**(9), D05109 (1998).

[50] Allen RG, Pereira LS, Howell TA, Jensen ME (2011). Evapotranspiration information reporting: I. Factors governing measurement accuracy. Agricult. Water Manage.

[51] Allen RG, Pereira LS (2009). Estimating crop coefficients from fraction of ground cover and height. Irrigation Sci.

[52] Wang Z, Liu Z, Zhang Z, Liu X (2009). Subsurface drip irrigation scheduling for cucumber (Cucumis sativus L.) grown in solar greenhouse based on 20cm standard pan evaporation in Northeast China. Sci. Hortic.

[53] Wang F, Kang S, Du T, Li F, Qiu R (2011). Determination of comprehensive quality index for tomato and its response to different irrigation treatments. Agricult. Water Manage.

[54] Fernández-Escobar R, Antonaya-Baena MF, Sánchez-Zamora MA, Molina-Soria C (2014). The amount of nitrogen applied and nutritional status of olive plants affect nitrogen uptake efficiency. Sci. Hortic.

[55] Olk DC (1998). Interpreting fertilizer-use efficiency in relation to soil nutrient-supplying capacity, factor productivity, and agronomic efficiency. Nutr. Cycl. Agroecosyst.

[56] Nin A, Arndt C, Hertel TW, Preckel PV (2003). Bridging the gap between partial and total factor productivity measures using directional distance functions. Am. J. Agric. Econ..

[57] Yadav RL (1998). Factor productivity trends in a rice–wheat cropping system under long-term use of chemical fertilizers. Exp. Agric.

[58] Shah K, Singh M, Rai AC (2015). Bioactive compounds of tomato fruits from transgenic plants tolerant to drought. LWT-Food Sci. Technol..

[59] Li T, Heuvelink EP, Marcelis LFM (2015). Quantifying the source–sink balance and carbohydrate content in three tomato cultivars. Front. Plant Sci..

[60] Ripoll J, Urban L, Bertin N (2015). The potential of the MAGIC TOM Parental accessions to explore the genetic variability in tomato acclimation to repeated cycles of water deficit and recovery. Front. Plant Sci..

[61] Watanabe M, Ohta Y, Licang S, Motoyama N, Kikuchi J (2015). Profiling contents of water-soluble metabolites and mineral nutrients to evaluate the effects of pesticides and organic and chemical fertilizers on tomato fruit quality. Food Chem..

[62] Mitchell JP, Shennan C, Grattan SR, May DM (1991). Tomato fruit yields and quality under water deficit and salinity. J. Am. Soc. Hortic. Sci..

[63] Jolliffe, I. *Principal component analysis* (Wiley Online Library, 2002).

[64] Richardson, M. *Principal component analysis* http://www.sdss.jhu.edu/~szalay/class/2015/SignalProcPCA.pdf (2009).

[65] Abdi H, Williams LJ (2010). Principal component analysis. Wiley Interdiscip. Rev. Comput. Stat.

