# Supplementary material for: Evaluation of the effects of irrigation and fertilization on tomato fruit yield and quality: a principal component analysis
Source: Sci Rep. 2017 Mar 23;7:350. doi: 10.1038/s41598-017-00373-8 (PMC5428234; doi:10.1038/s41598-017-00373-8)
Supplement: Supplementary file 1 — Supplementary Information [file 41598_2017_373_MOESM1_ESM.pdf]

Supplementary Information (SI)

Evaluation of the effects of irrigation and fertilization on tomato fruit yield and quality: a principal component analysis

Xiukang Wang[1, 2]* and Yingying Xing[1]*

1 College of Life Science, Yan'an University, Yan'an, Shaanxi 716000, China

2 State Key Laboratory of Soil Erosion and Dryland Farming on the Loess Plateau, Institute of Soil and Water Conservation, Chinese Academy of Sciences and Ministry of Water Resources, Yangling, Shaanxi 712100, China

* These authors made equal contribution, with Xiukang Wang conducted the principal component analysis and Yingying Xing conducted WUE, PFP, fruit quality and yield of tomatoes analysis but both contributed to the manuscript drafting.

Corresponding author: wangxiukang@126.com

Address: 580 Shengdi Road, Yan'an, Shaanxi 716000, China

Tel/Fax: +86 911 2332030

Email: wangxiukang@126.com

Supplematary material and methods

Supplement Tables: 15

Supplement Figure: 9

Supplementary Table S1. Analysis of variance summary for yields, water use efficiency (WUE) and partial factor productivity (PFP) as affected by irrigation (I) and fertilization treatment (F).

Item	Source	DF	MS	F value	Sign‡
Yield	Intercept	1	5.39E+11	2.57E+04	***
	Irrigation (I)	2	1.12E+09	688.81	***
	Fertilization (F)	2	9.14E+08	1.46E+03	***
	Replication (R)	8	2.10E+07	12.35	***
	I × F	4	7.68E+07	140.48	***
	I × R	16	1.62E+06	2.97	**
	F × R	16	6.25E+05	1.14	NS
	I × F × R	32	5.46E+05	–	–
WUE	Intercept	1	1.11E+05	1.58E+04	***
	Irrigation (I)	2	1.11E+03	664.22	***
	Fertilization (F)	2	168.35	1.39E+03	***
	Replication (R)	8	6.99	4.30	**
	I × F	4	8.13	51.04	***
	I × R	16	1.67	10.45	***
	F × R	16	0.12	0.76	NS
	I × F × R	32	0.16	–	–
PFP	Intercept	1	4.18E+06	2.37E+04	***
	Irrigation (I)	2	7.23E+03	646.28	***
	Fertilization (F)	2	1.08E+05	6.80E+03	***
	Replication (R)	8	176.31	7.56	***
	I × F	4	128.24	34.49	***
	I × R	16	11.19	3.01	**
	F × R	16	15.84	4.26	***
	I × F × R	32	3.72	–	–

‡"***" means $p < 0.001$, "**" means $0.001 < p < 0.01$, "*" means $0.01 < p < 0.05$ and "NS" means $p > 0.05$.

Supplementary Table S2. Effects of irrigation amount and fertilization levels on mean yields, water use efficiency (WUE) and partial factor productivity (PFP) in three consecutive growing seasons.

Treatment	Yield (10^3 kg ha^{-1})	WUE (kg m^{-3})	PFP (kg kg^{-1})
W1F1	95.8 ±1.56 a‡	34.5 ±0.61 de	187.9 ±3.06 f
W1F2	89.9 ±1.32 b	32.2 ±0.47 f	235.1 ±3.45 c
W1F3	78.0 ±1.43 e	27.6 ±0.49 g	305.8 ±5.61 a
W2F1	85.7 ±1.65 c	37.0 ±1.02 c	168.0 ±3.24 g
W2F2	83.0 ±2.06 d	36.1 ±1.11 cd	217.0 ±5.40 d
W2F3	76.2 ±1.71 e	33.2 ±0.63 ef	299.0 ±6.71 a
W3F1	78.0 ±1.91 e	45.3 ±1.86 a	153.0 ±3.74 h
W3F2	75.9 ±1.78 e	44.9 ±1.43 a	198.5 ±4.67 e
W3F3	71.2 ±2.17 f	41.7 ±1.49 b	279.2 ±8.49 b

W1: 100% ET_0, W2: 75% ET_0, W3: 50% ET_0, and F1: 240N−120P_2O_5−150K_2O kg ha^{-1}, F2: 180N−90P_2O_5−112.5K_2O kg ha^{-1}, F3: 120N−60P_2O_5−75K_2O kg ha^{-1}.

‡Values followed by a different lowercase letter between management treatments are significantly different $p < 0.05$ according to Tukey's HSD mean separation test.

Supplementary Table S3. Analysis of variance summary for total soluble solids (TSS), organic acid (OA) and Lycopene as affected by irrigation (I) and fertilization (F) treatment.

Item	Source	DF	MS	F value	Sign‡
TSS	Intercept	1	1.93E+03	4.17E+04	***
	Irrigation (I)	2	0.069	5.269	*
	Fertilization (F)	2	0.276	17.813	***
	Replication (R)	8	0.046	3.523	NS
	I × F	4	0.017	1.116	NS
	I × R	16	0.013	0.846	NS
	F × R	16	0.015	1.006	NS
	I × F × R	32	0.015	–	–
OA	Intercept	1	6.483	3.82E+03	***
	Irrigation (I)	2	0.006	12.137	**
	Fertilization (F)	2	0.031	46.899	***
	Replication (R)	8	0.002	1.829	NS
	I × F	4	0.001	3.157	*
	I × R	16	0.000	2.292	*
	F × R	16	0.001	3.038	**
	I × F × R	32	0.000	–	–
Lycopene	Intercept	1	1.08E+05	2.34E+03	***
	Irrigation (I)	2	2.24E+03	115.180	***
	Fertilization (F)	2	1.96E+03	134.877	***
	Replication (R)	8	45.934	1.823	NS
	I × F	4	29.847	3.387	*
	I × R	16	19.486	2.211	*
	F × R	16	14.518	1.647	NS
	I × F × R	32	8.813	–	–

‡"***" means $p < 0.001$, "**" means $0.001 < p < 0.01$, "*" means $0.01 < p < 0.05$ and "NS" means $p > 0.05$.

Supplementary Table S4. Analysis of variance summary for soluble sugar content (SSC) and vitamin C content (VC) as affected by irrigation (I) and fertilization (F) treatment.

Item	Source	DF	MS	F value	Sign‡
SSC	Intercept	1	694.666	2.84E+04	***
	Irrigation (I)	2	0.822	36.429	***
	Fertilization (F)	2	2.897	140.344	***
	Replication (R)	8	0.024	1.152	NS
	I × F	4	0.023	1.028	NS
	I × R	16	0.023	1.028	NS
	F × R	16	0.021	0.940	NS
	I × F × R	32	0.022	–	–
VC	Intercept	1	6.84E+04	2.30E+03	***
	Irrigation (I)	2	1.27E+03	194.969	***
	Fertilization (F)	2	222.626	76.320	**
	Replication (R)	8	29.744	4.336	*
	I × F	4	9.151	3.553	*
	I × R	16	6.518	2.530	*
	F × R	16	2.917	1.132	NS
	I × F × R	32	2.576	–	–

‡"***" means $p < 0.001$, "**" means $0.001 < p < 0.01$, "*" means $0.01 < p < 0.05$ and "NS" means $p > 0.05$.

Supplementary Table S5. Analysis of variance summary for nitrate concentration (NC) and sugar/acid content ratio (SAR) as affected by irrigation (I) and fertilization (F) treatment.

Item	Source	DF	MS	F value	Sign‡
NC	Intercept	1	2.01E+05	1.03E+04	***
	Irrigation (I)	2	752.897	90.902	***
	Fertilization (F)	2	1.76E+03	156.619	***
	Replication (R)	8	19.515	1.699	NS
	I × F	4	24.552	3.063	*
	I × R	16	8.283	1.033	NS
	F × R	16	11.218	1.400	NS
	I × F × R	32	8.015	–	–
SAR	Intercept	1	9.02E+03	9.45E+03	***
	Irrigation (I)	2	0.030	0.033	NS
	Fertilization (F)	2	0.975	0.611	NS
	Replication (R)	8	0.954	0.509	NS
	I × F	4	1.199	1.859	NS
	I × R	16	0.923	1.431	NS
	F × R	16	1.596	2.476	*
	I × F × R	32	0.645	–	–

‡"***" means $p < 0.001$, "**" means $0.001 < p < 0.01$, "*" means $0.01 < p < 0.05$ and "NS" means $p > 0.05$.

Supplementary Table S6. Mean vitamin C, soluble sugar content, total soluble solids, nitrate, lycopene, organic acid and sugar−acid ratio results from the different irrigation and fertilization levels on tomato fruit quality.

Treatment	TSS (%)	OA (%)	Lycopene (mg kg^{-1})	SSC (%)	VC (mg $100g^{-1}$)	NC (mg kg^{-1})	SAR
W1F1	4.94	0.29	33.41	3.03	25.07	51.73	10.74
W1F2	4.86	0.27	27.20	2.76	22.47	47.43	10.40
W1F3	4.67	0.23	20.43	2.43	20.16	37.94	10.49
W2F1	5.01	0.31	45.68	3.30	30.45	56.11	11.09
W2F2	4.87	0.30	38.44	3.01	28.48	47.15	10.19
W2F3	4.81	0.25	27.14	2.55	26.16	40.27	10.29
W3F1	5.01	0.34	54.28	3.40	40.31	64.35	10.35
W3F2	4.92	0.30	46.43	3.08	36.11	57.08	10.46
W3F3	4.84	0.26	34.93	2.79	32.28	45.75	10.96

Notes: TSS: total soluble solids, OA: organic acid, SSC: soluble sugar content, VC: vitamin C content, NC: nitrate concentration, SAR: sugar/acid content ratio.

Supplementary Table S7. The standardized values of tomato fruit quality.

Treatment	TSS (%)	OA (%)	Lycopene (mg kg^{-1})	SSC (%)	Vc (mg $100g^{-1}$)	NC (mg kg^{-1})	SAR
W1F1	0.54	0.37	−0.28	0.33	−0.62	0.23	0.61
W1F2	−0.24	−0.32	−0.85	−0.52	−1.02	−0.28	−0.50
W1F3	−2.01	−1.52	−1.47	−1.53	−1.37	−1.40	−0.19
W2F1	1.21	0.72	0.85	1.16	0.22	0.75	1.74
W2F2	−0.09	0.49	0.18	0.24	−0.09	−0.31	−1.17
W2F3	−0.66	−1.01	−0.85	−1.16	−0.45	−1.13	−0.85
W3F1	1.21	1.66	1.64	1.46	1.74	1.73	−0.65
W3F2	0.38	0.46	0.92	0.46	1.09	0.87	−0.30
W3F3	−0.35	−0.85	−0.14	−0.43	0.50	−0.47	1.31

Notes: TSS: total soluble solids, OA: organic acid, SSC: soluble sugar content, VC: vitamin C content, NC: nitrate concentration, SAR: sugar/acid content ratio.

Supplementary Table S8. The correlation matrix comes from the standardized values of tomato fruit quality.

Item	TSS (%)	OA (%)	Lycopene (mg kg^{-1})	SSC (%)	Vc (mg 100g^{-1})	NC (mg kg^{-1})	SAR
TSS (%)	1.00	0.92	0.86	0.95	0.69	0.91	0.28
TA (%)	0.92	1.00	0.94	0.98	0.76	0.94	0.01
Lycopene (mg kg^{-1})	0.86	0.94	1.00	0.94	0.92	0.94	0.13
SSC (%)	0.95	0.98	0.94	1.00	0.75	0.95	0.22
Vc (mg 100g^{-1})	0.69	0.76	0.92	0.75	1.00	0.81	0.06
NC (mg kg^{-1})	0.91	0.94	0.94	0.95	0.81	1.00	0.15
SAR	0.28	0.01	0.13	0.22	0.06	0.15	1.00

Notes: TSS: total soluble solids, OA: organic acid, SSC: soluble sugar content, VC: vitamin C content, NC: nitrate concentration, SAR: sugar/acid content ratio.

Supplementary Table S9. Total variance explained of the contribution rate and accumulative of contribution rate with eigenvalues was calculated by principal component analysis.

Component	Initial Eigenvalues			Extraction Sums of Squared Loadings			Rotation Sums of Squared Loadings		
	Total	% of Variance	Cumulative %	Total	% of Variance	Cumulative %	Total	% of Variance	Cumulative %
1	5.42	77.47	77.47	5.42	77.47	77.47	5.36	76.60	76.60
2	1.04	14.81	92.28	1.04	14.81	92.28	1.10	15.67	92.28
3	0.41	5.85	98.13						
4	0.07	0.99	99.12						
5	0.05	0.78	99.89						
6	0.01	0.11	100.00						
7	0.00	0.00	100.00						

Extraction Method: Principal Component Analysis.

Supplementary Table S10. The two components are extracted matrix by principal component analysis of fruit quality.

Item	Component	
	1	2
TSS (%)	0.94	0.14
OA (%)	0.95	−0.17
Lycopene (mg kg^{-1})	0.98	−0.06
SSC (%)	0.98	0.06
Vc (mg $100g^{-1}$)	0.85	−0.13
NC (mg kg^{-1})	0.97	−0.03
SAR	0.18	0.98

Notes: TSS: total soluble solids, OA: organic acid, SSC: soluble sugar content, VC: vitamin C content, NC: nitrate concentration, SAR: sugar/acid content ratio.

Supplementary Table S11. Mean vitamin C, soluble sugar content, total soluble solids, nitrate, lycopene, organic acid, sugar–acid ratio, yields, and WUE and PFP results from the different irrigation and fertilization levels on tomato fruit.

Treatment	Yields (kg ha^{-1})	WUE (kg m^{-3})	PFP (kg kg^{-1})	TSS (%)	TA (%)	Lycopene (mg kg^{-1})	SSC (%)	VC (mg 100g^{-1})	NC (mg kg^{-1})	SAR
W1F1	95825.29	34.54	187.89	4.94	0.29	33.41	3.03	25.07	51.73	10.74
W1F2	89933.27	32.20	235.12	4.86	0.27	27.20	2.76	22.47	47.43	10.40
W1F3	77980.70	27.56	305.81	4.67	0.23	20.43	2.43	20.16	37.94	10.49
W2F1	85681.65	37.04	168.00	5.01	0.31	45.68	3.30	30.45	56.11	11.09
W2F2	83002.27	36.11	217.00	4.87	0.30	38.44	3.01	28.48	47.15	10.19
W2F3	76244.95	33.21	299.00	4.81	0.25	27.14	2.55	26.16	40.27	10.29
W3F1	78044.01	45.30	153.03	5.01	0.34	54.28	3.40	40.31	64.35	10.35
W3F2	75917.62	44.91	198.48	4.92	0.30	46.43	3.08	36.11	57.08	10.46
W3F3	71207.72	41.69	279.25	4.84	0.26	34.93	2.79	32.28	45.75	10.96

Notes: TSS: total soluble solids, OA: organic acid, SSC: soluble sugar content, VC: vitamin C content, NC: nitrate concentration, SAR: sugar/acid content ratio.

Supplementary Table S12. The standardized values of yield, WUE, PFP and tomato fruit quality.

Treatment	Yields ($kg\ ha^{-1}$)	WUE ($kg\ m^{-3}$)	PFP ($kg\ kg^{-1}$)	TSS (%)	TA (%)	Lycopene ($mg\ kg^{-1}$)	SSC (%)	VC ($mg\ 100g^{-1}$)	NC ($mg\ kg^{-1}$)	SAR
W1F1	1.83	−0.40	−0.69	0.54	0.37	−0.28	0.33	−0.62	0.23	0.61
W1F2	1.08	−0.79	0.14	−0.24	−0.32	−0.85	−0.52	−1.02	−0.28	−0.50
W1F3	−0.46	−1.57	1.39	−2.01	−1.52	−1.47	−1.53	−1.37	−1.40	−0.19
W2F1	0.53	0.02	−1.04	1.21	0.72	0.85	1.16	0.22	0.75	1.74
W2F2	0.19	−0.14	−0.18	−0.09	0.49	0.18	0.24	−0.09	−0.31	−1.17
W2F3	−0.68	−0.62	1.27	−0.66	−1.01	−0.85	−1.16	−0.45	−1.13	−0.85
W3F1	−0.45	1.39	−1.31	1.21	1.66	1.64	1.46	1.74	1.73	−0.65
W3F2	−0.72	1.33	−0.51	0.38	0.46	0.92	0.46	1.09	0.87	−0.30
W3F3	−1.33	0.79	0.92	−0.35	−0.85	−0.14	−0.43	0.50	−0.47	1.31

Notes: WUE: water use efficiency, PFP: partial factor productivity, TSS: total soluble solids, OA: organic acid, SSC: soluble sugar content, VC: vitamin C content, NC: nitrate concentration, SAR: sugar/acid content ratio.

Supplementary Table S13. The correlation matrix comes from the standardized values of yield, WUE, PFP and tomato fruit quality.

Item	Yields (kg ha^{-1})	WUE (kg m^{-3})	PFP (kg kg^{-1})	TSS (%)	TA (%)	Lycopene (mg kg^{-1})	SSC (%)	Vc (mg 100g^{-1})	NC (mg kg^{-1})	SAR
Yields (kg ha^{-1})	1.00	−0.39	−0.43	0.29	0.28	−0.11	0.22	−0.43	0.14	0.09
WUE (kg m^{-3})	−0.39	1.00	−0.57	0.69	0.66	0.88	0.71	0.97	0.78	0.11
PFP (kg kg^{-1})	−0.43	−0.57	1.00	−0.92	−0.97	−0.84	−0.96	−0.60	−0.93	−0.16
TSS (%)	0.29	0.69	−0.92	1.00	0.91	0.87	0.95	0.70	0.91	0.28
TA (%)	0.28	0.66	−0.97	0.91	1.00	0.90	0.97	0.71	0.94	−0.02
Lycopene (mg kg^{-1})	−0.11	0.88	−0.84	0.87	0.90	1.00	0.94	0.92	0.93	0.13
SSC (%)	0.22	0.71	−0.96	0.95	0.97	0.94	1.00	0.75	0.95	0.23
Vc (mg 100g^{-1})	−0.43	0.97	−0.60	0.70	0.71	0.92	0.75	1.00	0.81	0.06
NC (mg kg^{-1})	0.14	0.78	−0.93	0.91	0.94	0.93	0.95	0.81	1.00	0.15
SAR	0.09	0.11	−0.16	0.28	−0.02	0.13	0.23	0.06	0.15	1.00

Notes: WUE: water use efficiency, PFP: partial factor productivity, TSS: total soluble solids, OA: organic acid, SSC: soluble sugar content, VC: vitamin C content, NC: nitrate concentration, SAR: sugar/acid content ratio.

Supplementary Table S14. Total variance explained of the contribution rate and accumulative of contribution rate with eigenvalues was calculated by principal component analysis.

Component	Initial Eigenvalues			Extraction Sums of Squared Loadings			Rotation Sums of Squared Loadings		
	Total	% of Variance	Cumulative %	Total	% of Variance	Cumulative %	Total	% of Variance	Cumulative %
1	6.96	69.61	69.61	6.96	69.61	69.61	6.75	67.53	67.53
2	1.79	17.85	87.47	1.79	17.85	87.47	1.93	19.27	86.80
3	1.02	10.17	97.63	1.02	10.17	97.63	1.08	10.83	97.63
4	0.12	1.17	98.81						
5	0.08	0.76	99.56						
6	0.03	0.34	99.90						
7	0.01	0.08	99.98						
8	0.00	0.02	100.00						
9	0.00	0.00	100.00						
10	(0.00)	(0.00)	100.00						

Extraction Method: Principal Component Analysis.

Supplementary Table S15. The three components extracted matrix by principal component analysis of yield, WUE, PFP and tomato fruit quality.

Item	Component		
	1	2	3
Yields (kg ha^{-1})	0.09	0.98	−0.09
WUE (kg m^{-3})	0.83	−0.50	0.06
PFP (kg kg^{-1})	−0.92	−0.36	0.07
TSS (%)	0.94	0.22	0.09
OA (%)	0.95	0.19	−0.22
Lycopene (mg kg^{-1})	0.97	−0.19	−0.01
SSC (%)	0.98	0.16	0.03
Vc (mg $100g^{-1}$)	0.85	−0.52	0.00
NC (mg kg^{-1})	0.98	0.05	−0.03
SAR	0.17	0.17	0.97

Notes: WUE: water use efficiency, PFP: partial factor productivity, TSS: total soluble solids, OA: organic acid, SSC: soluble sugar content, VC: vitamin C content, NC: nitrate concentration, SAR: sugar/acid content ratio.

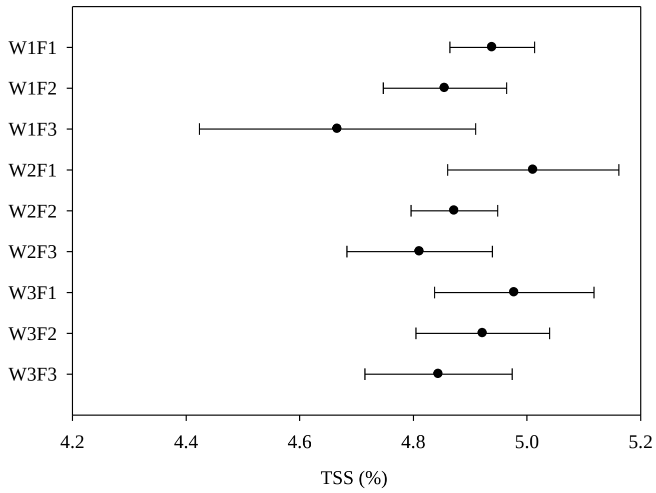

Supplementary Figure S1. The effect of irrigation and fertilization on total soluble solids (TSS) at different water and fertilizer input levels. Data are means of nine replicates, which was composed of three consecutive growing seasons and three replications per treatments and displayed as low and high (from top to the bottom).

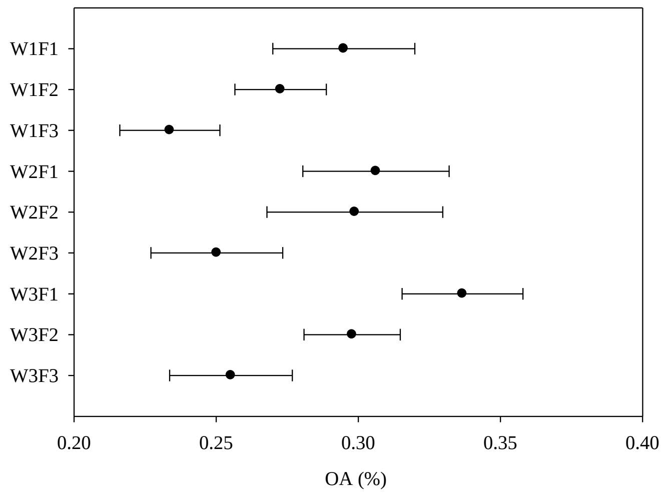

Supplementary Figure S2. The effect of irrigation and fertilization on organic acid (OA) at different water and fertilizer input levels. Data are means of nine replicates, which was composed of three consecutive growing seasons and three replications per treatments and displayed as low and high (from top to the bottom).

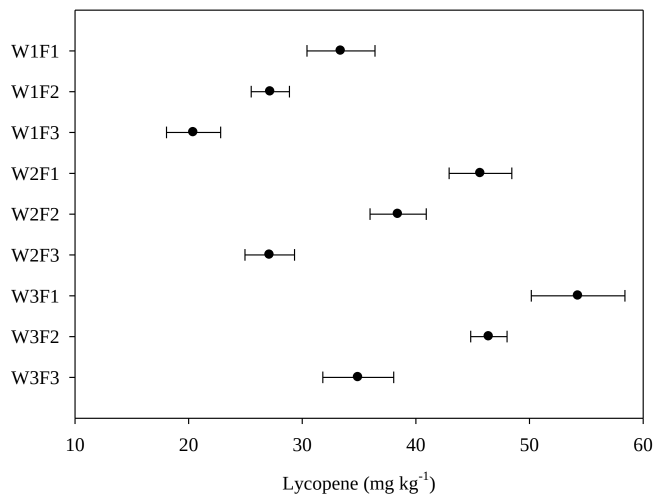

Supplementary Figure S3. The effect of irrigation and fertilization on lycopene at different water and fertilizer input levels. Data are means of nine replicates, which was composed of three consecutive growing seasons and three replications per treatments and displayed as low and high (from top to the bottom).

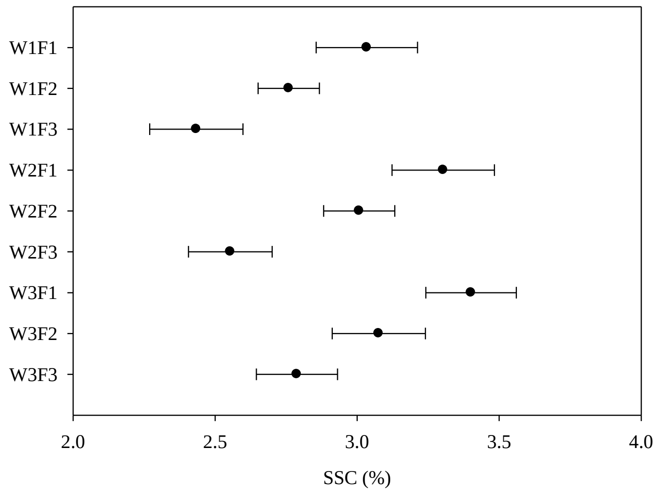

Supplementary Figure S4. The effect of irrigation and fertilization on soluble sugar content (SSC) at different water and fertilizer input levels. Data are means of nine replicates, which was composed of three consecutive growing seasons and three replications per treatments and displayed as low and high (from top to the bottom).

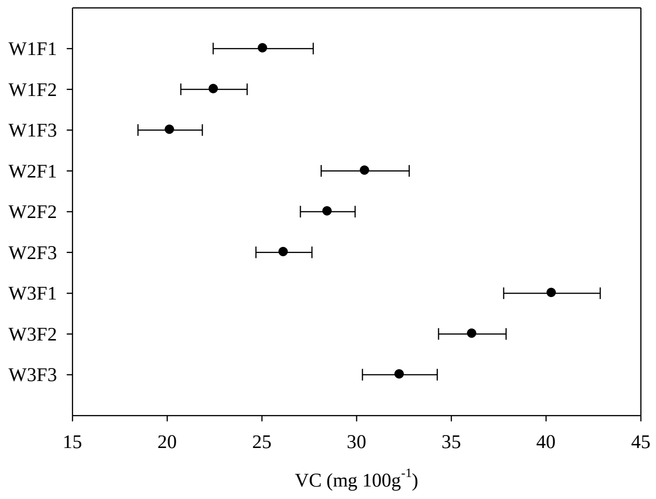

Supplementary Figure S5. The effect of irrigation and fertilization on vitamin C (VC) at different water and fertilizer input levels. Data are means of nine replicates, which was composed of three consecutive growing seasons and three replications per treatments and displayed as low and high (from top to the bottom).

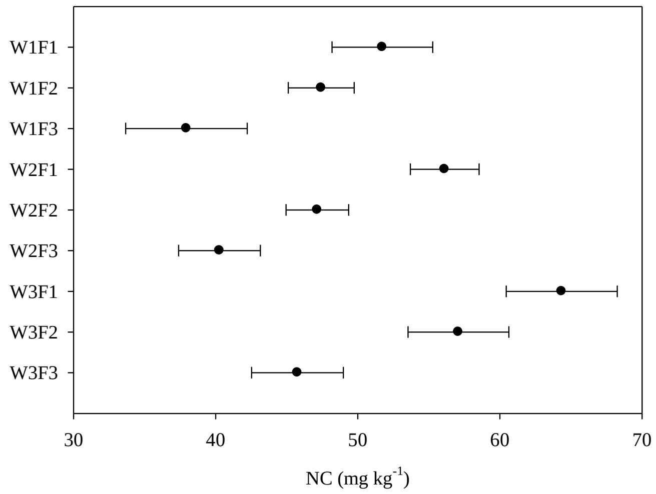

Supplementary Figure S6. The effect of irrigation and fertilization on nitrate concentration (NC) at different water and fertilizer input levels. Data are means of nine replicates, which was composed of three consecutive growing seasons and three replications per treatments and displayed as low and high (from top to the bottom).

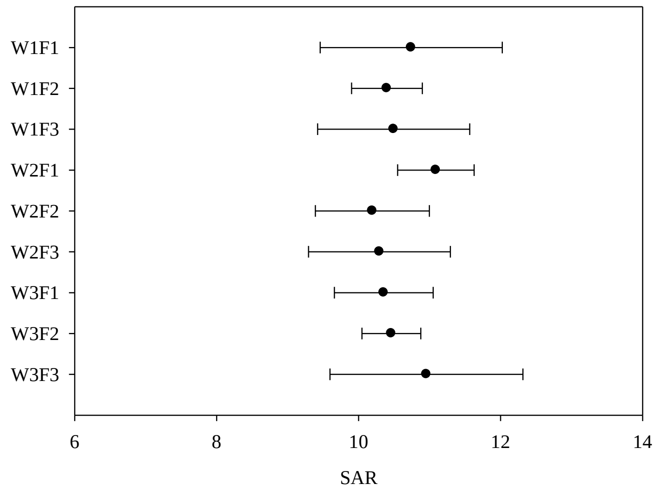

Supplementary Figure S7. The effect of irrigation and fertilization on sugar/acid content ratio (SAR) at different water and fertilizer input levels. Data are means of nine replicates, which was composed of three consecutive growing seasons and three replications per treatments and displayed as low and high (from top to the bottom).

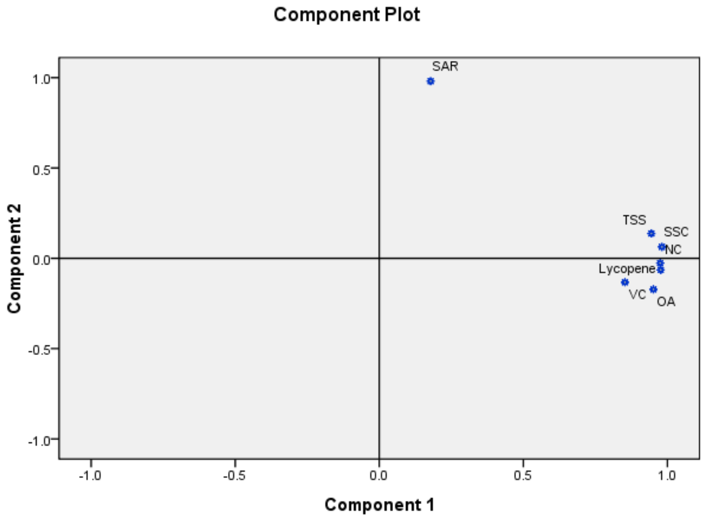

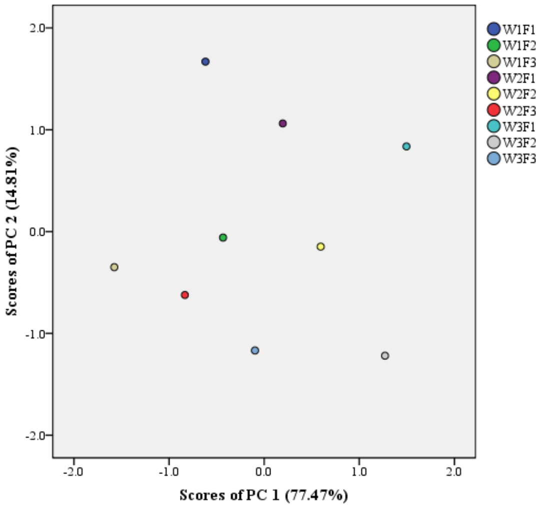

Supplementary Figure S8. Principal components analysis (PCA) plots and Score plot of a two component PCA model of fruit quality. Notes: TSS: total soluble solids, OA: organic acid, SSC: soluble sugar content, VC: vitamin C content, NC: nitrate concentration, SAR: sugar/acid content ratio.

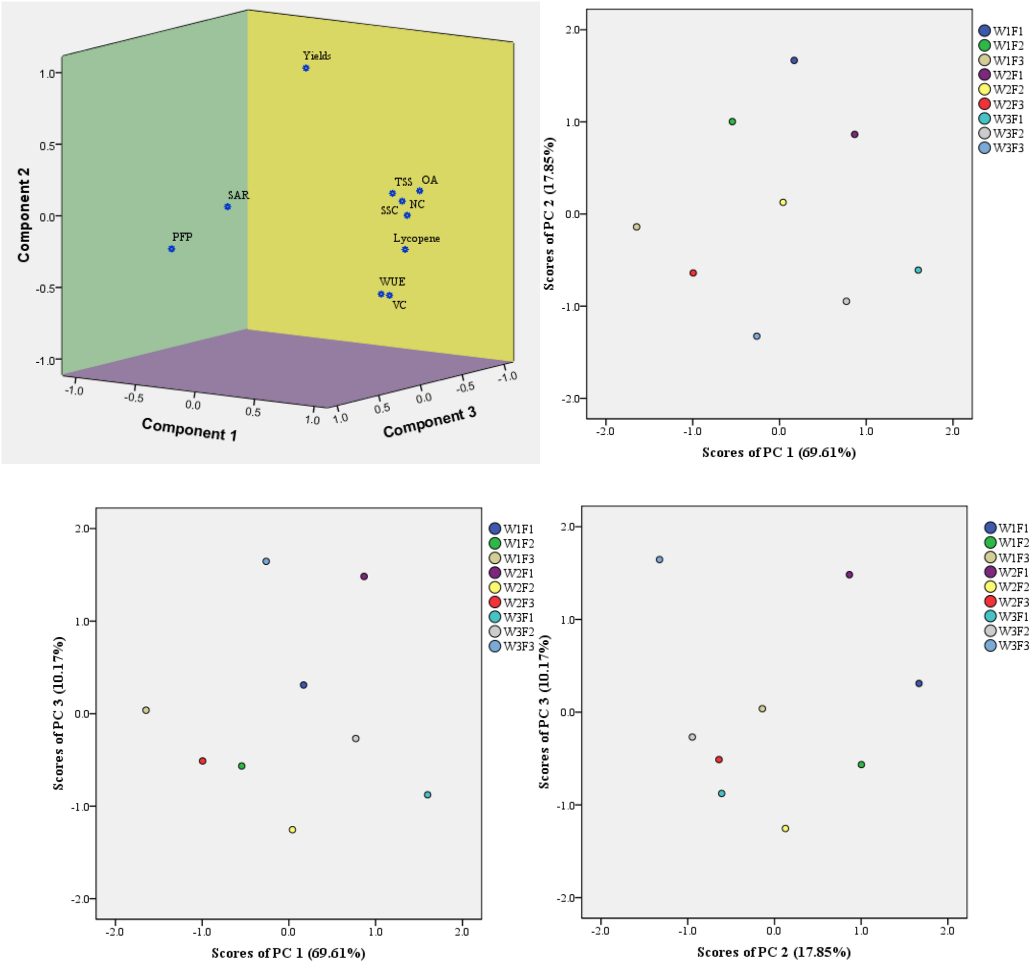

Supplementary Figure S9. Principal components analysis (PCA) plots and score plot of a three component PCA model of yield, WUE, PFP and tomato fruit quality. Notes: WUE: water use efficiency, PFP: partial factor productivity, TSS: total soluble solids, OA: organic acid, SSC: soluble sugar content, VC: vitamin C content, NC: nitrate concentration, SAR: sugar/acid content ratio.
